# Comparison of the stabilized waste soil properties and stabilization mechanism of phosphogypsum-fly ash-steel slag based cement versus Portland cement

**DOI:** 10.1371/journal.pone.0318862

**Published:** 2025-06-03

**Authors:** Jianmin Guo, Xizhong Xu, Xiaohui Song, Jincheng Wei, Wencheng Shi, Yu Xia

**Affiliations:** 1 Shandong Hi-Speed Company Limited, Jinan, China; 2 Shandong Transportation Institute, Jinan, China; Shandong University of Technology, CHINA

## Abstract

Through physical and chemical reactions in the presence of phosphogypsum, steel slag and fly ash modify the load-bearing skeleton and fill the pores of the wasted soil, resulting in high-strength performance. Extensive experiments that compared Portland cement with phosphogypsum-fly ash-steel slag-based cement (PFS cement) revealed that the later-stage unconfined compressive strength (UCS) of PFS cement exceeded that of Portland cement by approximately 33.18%. In terms of the stress-strain curve, the maximum stress and yield strength of PFS cement-stabilized waste soil were 40% to 50% higher than those of Portland cement. In the dry-wet cycle resistance experiment, PFS cement-stabilized waste soil showed a compressive strength increase of 15.17% over Portland cement. Furthermore, during the freeze-thaw cycle test, PFS cement-stabilized waste soil demonstrated a 29.95% higher performance compared to Portland cement. When used as a solidifying agent, PFS cement exhibits significant advantages over Portland cement in backfilling for underground engineering, trenches, roadbeds, bridge abutments, and road base layers.

## 1. Introduction

Engineering waste soil is classified as construction waste, posing numerous hazards to the environment and society. It poses a significant obstacle to urban development. Harmful substances, such as heavy metals and chemical pollutants, are typically present in large amounts in engineering waste soil [1]. Soil and groundwater can be severely contaminated by these substances through infiltration and diffusion [2]. During storage, waste soil generates dust and harmful gases, which threaten human health [3,4], especially by increasing the risk of respiratory diseases. Additionally, direct backfilling or storing of engineering waste soil in the natural environment damages soil structure [5]. Direct backfilling or storing of engineering waste soil affects vegetation growth and reduces crop yield. Large-scale storage of waste soil alters surface and subsurface hydrological cycles [6]. It also obstructs surface runoff and increases flood risks [7]. Proper treatment of engineering waste soil is urgently needed.

The optimal approach to managing engineering waste soil involves resource reuse, effectively stabilizing waste soil to facilitate its reuse. Methods for soil stabilization are continuously evolving. A cementing solution with an ion concentration of 0.75 M and a urea concentration of 1.0 M, combined with a bacterial suspension, effectively stabilizes loess particles and enhances the collapse resistance of loess [8]. Using fibers with a content of 0.3% and a length of 19 mm, combined with hard alloy slag for clay stabilization, significantly enhances the compressive and indirect tensile strengths of stabilized soil [9]. Marine soft clay stabilized with carbide slag and plant ash shows improved compressive strength [10]. The use of lignin and sodium silicate as additives increases the strength and cohesion of composite stabilized soil by 1.5 times and doubles its impermeability [11]. Among various treatment methods, physical stabilization proves to be insignificant, while biological treatment remains prohibitively expensive. Cement binders emerge as the most economical and effective solution for stabilizing soil intended for use as fluidized backfill material.

Incorporating Portland cement into soil triggers physical, chemical, and physico-chemical reactions. These reactions produce new solid-phase crystalline substances, which form a strong and stable spatial network structure within the soil. Structural strength significantly improves, achieving the goal of stabilizing waste soil. Combined with mechanical action, these reactions yield the best results. Wu used cement to stabilize marine soft soil and validated, through physical performance tests, compaction tests, and unconfined compressive strength tests, that Portland cement significantly improves the physical and mechanical properties of the soil [12]. Deng used cement-metakaolin composite materials to stabilize marine soft clay, experimentally verifying that metakaolin inhibits freeze-thaw cracking of stabilized soil [13]. Liang used cement and sand composite materials to stabilize South China coastal soft clay. The increase in sand content and reduction in sand particle size, as validated through unconfined compressive strength tests and dry-wet cycle tests, significantly improve the strength and durability of stabilized soil [14]. Cement-stabilized soil remains one of the core technologies for stabilizing waste soil.

The production of Portland cement requires significant resource consumption and energy demand, making it challenging to meet modern sustainable development requirements. Solid waste-based cement has notable advantages in reducing carbon emissions. This type of cement demonstrates excellent performance in soil stabilization, significantly improving soil stability, bearing capacity, mechanical properties, and durability. Desulfurized gypsum and slag composite gel materials show trends in unconfined compressive strength similar to those of cement-stabilized soil, meeting stabilization requirements [15]. Wei used sodium silicate and gypsum as a 12.64% composite additive to stabilize silt soil. Experimental results showed that the SG composite stabilizer outperformed cement-stabilized soil in impermeability, freeze-thaw resistance, and dry-wet resistance [16]. Wu used carbide slag, blast furnace slag, and fly ash to form a CGF all-solid waste alkali-activated binder to stabilize waste soil. The unit strength cost of stabilized soil is found to be 3.53-5.88 times that of the soil itself [17]. Solid waste-based cement becomes an important breakthrough in soil stabilization technology due to its environmental benefits and superior soil improvement performance.

In this research, industrial waste soil from Yantai City, Shandong Province, was selected as the research object to explore the effectiveness of using Portland cement and Phosphogypsum-fly ash-steel slag cement (PFS cement) as soil stabilizers for fluidized backfill material. The research focused on evaluating mechanical properties and durability. The research objectives include: (1) determining the optimal water-to-solid ratio for PFS cement stabilized soil used as fluidized backfill material through experiments; (2) to compare the mechanical properties of waste soil stabilized with PFS cement and Portland cement, and to evaluate the performance advantages of PFS cement; (3) evaluating and verifying the durability of PFS cement stabilized soil, exploring its improvements compared to Portland cement stabilized soil. The expected outcomes of this research will provide new solutions for stabilizing waste soil, help reduce carbon emissions, increase waste resource utilization, and achieve significant economic and social benefits.

## 2. Materials

### 2.1 Construction waste soil

**Fig 1:**
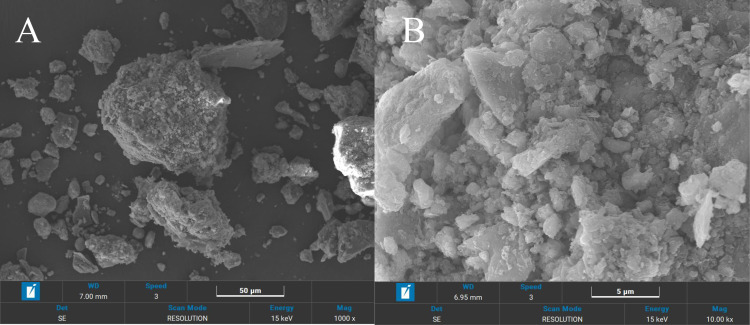
SEM analysis results of waste soil.

The soil samples utilized in this study were sourced from construction waste soil generated during the building development process in Yantai, Shandong Province. Approximately 300 cubic meters of waste soil had been stockpiled outdoors within the construction area, subject to prolonged exposure to the natural environment. This resulted in severe dust pollution, which adversely impacted the urban aesthetics and ecological environment, and led to the contamination of water bodies and air. Atterberg limits tests were performed using ASTM D4318 test method [18–20]. Based on the results, the liquid and plastic limits of soil were equal to 26.5% and 18.6%, respectively. According to the Unified classification as standardized in ASTM D2487 [21], this soil was classified as MH. Compaction test was performed using the modified proctor method according to ASTM D1557 [22]. Based on the obtained results the optimum moisture content and the maximum dry density of studied soil were equal to 16.4% and 1.74 g/cm3, respectively. The chemical composition of clay was determined using X-ray fluorescence spectroscopy (XRF) [23,24] method according to ASTM E1621 [25]. Table 1 presents the results of XRF test. Given this table, the main chemical compositions of studied clay consisted of SiO_2_, Al_2_O_3_, Fe_2_O_3_ and CaO. Further examination using scanning electron microscopy (SEM) [26,27] revealed that the Waste Soil exhibited a typical amorphous structure (Fig 1).

**Table 1:** XRF analysis results of waste soil.

CaO	SO_3_	Al_2_O_3_	SiO_2_	TiO_2_	Fe_2_O_3_	MgO	K_2_O	P_2_O_5_	Na_2_O	MnO	Others
20.6	1.16	11.64	49.71	0.77	7.31	4.65	2.16	0.21	1.39	0.17	0.24

### 2.2 Phosphogypsum-fly ash-steel slag cement

The study conducted a mix design for bulk solid waste materials such as coal gangue, lime, and wood ash. Comprehensive experimental results indicated that a combination of phosphogypsum, fly ash, and steel slag could effectively serve as a soil stabilizer, meeting high stability requirements. PFS cement is a low-carbon, ultra-slow-setting polymer material containing steel slag micropowder. It utilizes low-carbon preparation technology, combining gypsum slag-based binders and steel slag binders to produce PFS cement. Phosphogypsum, an industrial solid waste, serves as a conditioner to improve physical and chemical properties of soil. It slows soil degradation, immobilizes heavy metals, and substitutes natural gypsum for resource utilization. Fly ash, a major admixture for producing fluidized recycled backfill material, enhances flow properties. It also improves anti-segregation and anti-bleeding properties of recycled backfill material. Furthermore, it increases water stability and bandwidth. Activated steel slag increases compressive strength, acts as a water reducer, and minimizes expansion. It compensates for concrete shrinkage. The low-carbon preparation technology eliminates high-temperature sintering of cement clinker. This process significantly reduces carbon emissions during production.

### 2.3 Portland cement

This research used Portland cement, one of the most widely used types of cement. Portland cement is available as a fine gray or white powder. It is primarily composed of calcium silicates, aluminates, and oxides of iron and aluminum. Based on composition and application, Portland cement is classified into four types: calcareous, siliceous, aluminous, and ferrous [28]. When mixed with water, Portland cement undergoes a series of complex chemical reactions that produce an extremely strong substance. Hydration, the process at the core of cement applications in construction and engineering, makes this possible. To evaluate the performance of Portland cement in soil stabilization and compare it with PFS cement, this research conducted X-ray fluorescence (XRF) analysis on Portland cement.

## 3. Experiment

### 3.1 Flow performance experiment

#### 3.1.1 Mixing method of samples.

**Fig 2:**
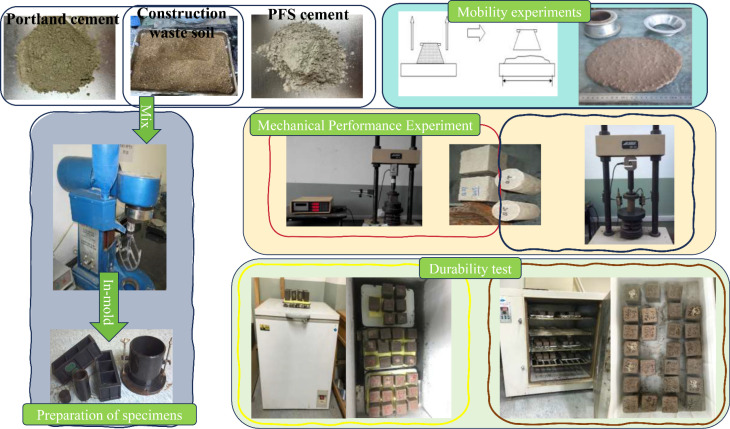
Performance testing experiment.

The stabilized soil was mixed using a planetary cement mortar mixer, with the mixing volume being approximately one-third of the total volume of the mixing container. First, the engineering waste soil was dried to a constant weight. The actual amounts of engineering waste soil, PFS cement, and water were calculated based on the total volume of the mixing container and placed into the bowl. The bowl was then placed on the fixed rack and raised to the fixed position. The machine was started immediately, and the dry soil was mixed for 60 seconds, followed by the addition of distilled water and further mixing for 280 seconds. The resulting stabilized waste soil was then subjected to performance testing. After obtaining the stabilized waste soil, experiments were conducted according to the specific requirements of each test (Fig 2). In the experiment, a cement dosage of 15% was selected. The dosage determination experiment included gradient levels of 9%, 12%, 15%, and 18%. Dosages above 18% were not chosen because the high engineering costs led to a lack of practical value. Experimental results indicated that stabilizers with dosages of 9% and 12% were unable to solidify the soil, whereas both 15% and 18% exhibited good fluidity and early solidification performance. Therefore, the lower-cost dosage of 15% was adopted as the experimental scheme.

#### 3.1.2 Fluidity experiment.

Fluidity is a key indicator for evaluating the workability of flowable backfill materials made from construction waste. Due to the lack of unified standards and regulations for fluidity testing of backfill materials in China, this study adopted the truncated cone method from T0507—2005 “Test Method for Determination of Fluidity of Cement Mortar”. In this study, PFS cement was used to determine the most suitable water-to-solid ratio for stabilizing engineering waste soil. After determining the dosage of the soil stabilizer, an experiment was conducted using PFS cement to determine the optimal liquid-to-solid ratio. Five mixture ratios were set: 0.35, 0.39, 0.43, 0.47, and 0.51, allowing for repeatable experiments.

After mixing the stabilized soil according to the specified ratios, it was poured into the measuring tool without compaction. After smoothing the surface, the mold was lifted vertically. Once the expansion of the stabilized soil reached a stable state, the maximum spread diameter and its perpendicular diameter were measured. The average of these two measurements was taken as the flow value of the stabilized soil.

### 3.2 Mechanical performance experiment

#### 3.2.1 Compressive strength experiment.

The compressive strength test referred to the T 0553-2005 standard in the “Test Methods of Cement and Concrete for Highway Engineering” (JTG E30-2005). The sample preparation method was the same as in the fluidity experiment. After mixing, the stabilized soil was placed into molds and cured in a constant temperature chamber until the specified age. Before conducting the compressive strength test, the surface of the specimen was cleaned, and its dimensions were measured to an accuracy of 1 mm. If the difference between the measured dimensions and the nominal dimensions was within 1 mm, the nominal dimensions were used for calculations. The specimen was placed on the lower platen or base plate of the testing machine, ensuring that the load-bearing surface was perpendicular to the top surface formed during molding and that the center of the specimen aligned with the center of the lower platen or base plate. The testing machine was started. When the upper platen approached the specimen or upper base plate, the ball seat was adjusted to ensure even pressure on the contact surface. The compressive test was performed continuously and uniformly at a loading rate of 1 mm/min until the specimen failed, and the failure load was recorded. Laboratory mix design strength tests were conducted for PFS cement and Portland cement. Two groups of tests were designed, each containing 18 specimens. Raw materials included waste soil, PFS cement, and Portland cement. Experiment factors involved a lime-soil ratio of 0.15 and a liquid-solid ratio of 0.43. Specimens were formed and cured, and unconfined compressive strength was measured at six curing ages: 7 days, 28 days, 60 days, 75 days, 90 days, and 120 days. Three specimens were tested at each age, and the average value was calculated. Non-representative specimens were supplemented with additional tests. The experimental scheme is shown in Table 2.

**Table 2:** Unconfined compressive strength test plan.

Category	Age (days)					
	7d	28d	60d	75d	90d	120d
	Number of Tests					
PFS cement	3	3	3	3	3	3
Portland cement	3	3	3	3	3	3

#### 3.2.2 Stress-strain experiment.

The stress-strain test was used to evaluate the stiffness characteristics of stabilized soil. Based on improvements to the compressive strength test, a testing method for stress-strain behavior of recycled backfill materials was developed to determine the stress-strain relationship of these materials. The stress-strain test was conducted using an LD133 pavement material strength tester, which includes a DHDAS dynamic signal acquisition [29] and analysis system for real-time measurement of axial load and axial deformation [30,31]. Alternatively, the test could also be performed using a UTM universal testing machine [32]. To carry out the test, various instruments and gauges were set up, with two micrometers placed at different positions to measure specimen deformation. The average deformation value from both micrometers was used for calculations. The platform was gradually raised, and just before the specimen made contact with the pressure plate, the micrometers were zeroed. The platform was then loaded at a speed of 1-1.25 mm/min, and all deformation and pressure data were automatically collected by the instrument. The stress-strain calculation formula was given as follows (Eq. 1, Eq. 2). The deformation of the specimen (Y), strain $\left(\varepsilon \right)$ , applied pressure (F), stress (σ).


ε=Y/100 (1)

$$\sigma =4F/\left(\pi *50*50\right)$$ (2)

### 3.3 Durability performance experiment

Stabilized waste soil is mainly used for backfilling in underground engineering, trenches, roadbeds, abutments, and road bases. It is subjected to harsh environments such as dry-wet cycles and freeze-thaw cycles. Therefore, this comparative experiment included dry-wet cycle resistance and freeze-thaw cycle resistance tests. Two groups of tests were conducted for mixture designs involving PFS cement and Portland cement, as shown in Table 3. In the wet-dry cycle test, each specimen underwent 12 cycles, with unconfined compressive strength testing conducted after each cycle. In the freeze-thaw cycle test, each specimen also underwent 12 cycles, but testing was conducted only after the 4^th^, 8^th^, and 12^th^ cycles. Unconfined compressive strength was measured for three specimens at each interval, and the average value was calculated for the result. The compressive strength test for specimens after curing used the method outlined in Section 3.2.1. During durability tests, no specimen experienced cracking throughout the cycles.

**Table 3:** Durability test plan.

Category	admixture-Soil Ratio	Liquid-Solid Ratio	Number of Specimens	
			Wet-Dry Cycle	Freeze-Thaw Cycle
PFS cement	0.15	0.43	36	9
Portland cement	0.15	0.43	36	9

#### 3.3.1 Dry-wet cycle resistance experiment.

Based on ASTM D559-03, “Standard Test Methods for Wetting and Drying Compacted Soil-Cement Mixtures,” a wet-dry cycle test was designed specifically for stabilizing waste soil. After preparing the ds. The specimens were then cured for 28 days in a standard incubator before starting the cycles. stabilized material according to the mixture design, specimens were poured into mol Wet-dry cycles involved two parts: soaking and drying. Specimens were soaked at room temperature in distilled water at 32°C for 5 hours, then removed and dried in an oven at 71°C for 43 hours. This completed one cycle. The process was repeated for a total of 12 cycles. If a specimen failed during the cycles, the test ended early. After each cycle, unconfined compressive strength tests were performed on the specimens.

#### 3.3.2 Freeze-thaw cycle resistance experiment.

Following ASTM D560-03, “Standard Test Methods for Freezing and Thawing Compacted Soil-Cement Mixtures,” a freeze-thaw cycle test was designed to evaluate the performance of waste soil stabilized with cement. The stabilized material was prepared following the mixture design and then poured into molds. Specimens were cured for 27 days in a standard incubator. After curing, specimens were soaked at room temperature in distilled water at 32°C for 24 hours. The water level was kept at least 2 cm above the top surface of the specimens. After soaking, freeze-thaw cycles began, involving freezing and thawing phases. The soaked specimens were placed in trays with 2-3 cm sponge pads at the bottom. Water was then added until the base of the specimens just touched the water, ensuring the sponge pads were saturated. The tray, containing the specimens, was placed in a freezer to initiate the freezing phase. Specimen spacing was at least 5 cm horizontally and 25 cm vertically. Specimens were frozen at -10 ± 5°C for 12 hours (time required to reach this temperature was not included). After freezing, specimens and trays were placed in a controlled temperature and humidity room for thawing for 12 hours. After completing the above operations, one freeze-thaw cycle was finished. The process was repeated for a total of 12 cycles. If a specimen failed during the cycles, the test ended early. Compressive strength tests were performed on the specimens after every 4th cycle.

### 3.4 Analysis of soil stabilizing admixtures components

#### 3.4.1 XRF experiment.

The experiment used a PANalytical AXIOS X-ray fluorescence (XRF) spectrometer for quantitative multi-element analysis. The instrument was equipped with an advanced SST-mAX tube, combined with ZETA technology to eliminate drift and ensure stable performance throughout the tube’s lifespan. A CHI-BLUE coating reduced corrosion, improving tube performance. The experiment was conducted in a controlled environment with temperatures ranging from 10-35°C, maintaining stability at ≤±0.50°C. The power requirement was 220V ± 10%, allowing a fluctuation range of ± 15% to -10%. A low-noise high-voltage generator with a digital KV/mA display ensured output fluctuation at ≤±0.005%, even with external power variations of ± 10%. Analysis was performed using the downward irradiation method, requiring minimal or no sample preparation, allowing efficient automated analysis. Additionally, a split-type circulating cooling water system provided a maximum cooling capacity of 8 kW, maximum water pressure of 5 kg/cm², and a flow rate of 20 L/min, ensuring stable operation of the equipment.

## 4. Results

### 4.1 Fluidity test results

According to the 229r-99 standard for controlled low-strength materials, fluidized backfill materials were classified into three categories based on soil stability and fluidity levels [20]. Low fluidity materials, with a flow range of 160-180 mm, are suitable for backfilling larger spaces such as pipe trenches and roadbeds. General fluidity materials, with a flow range of 180-220 mm, are suitable for general backfilling projects. High fluidity materials, with a flow range greater than 220 mm, are suitable for backfilling narrow operation spaces or areas with dead angles. The experiment set up three repeat tests. According to the test results (refer to the table 4), when the water-to-solid ratio was 0.43, the fluidity of PFS cement stabilized soil was around 205 mm, showing the best fluidity. Therefore, the water-to-solid ratio used in the performance tests was 0.43.

**Table 4:** Fluidity test results.

Experimental Group	Flow Value/mm				
	0.35	0.39	0.43	0.47	0.51
G 1	174	186	206	219	232
G 2	180	191	202	221	229
G 3	173	193	208	216	229
Average Value	175.67	190.00	205.33	218.67	230.00

### 4.2 Results of mechanical performance

#### 4.2.1 Unconfined compressive strength.

**Fig 3:**
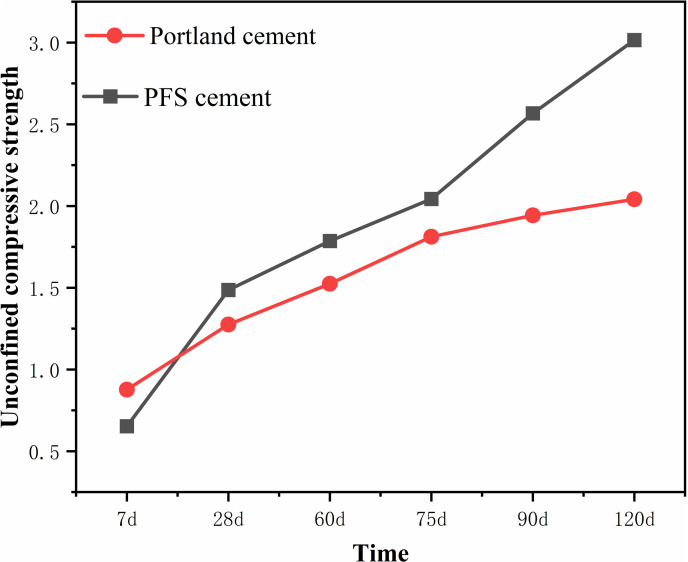
Unconfined compressive strength.


The strength of stabilized waste soil increased with curing age, reached a critical value, and then stabilized. To investigate the long-term strength growth pattern of stabilized waste soil, a compressive strength test was designed. The test used a water-to-solid ratio of 0.43 and a soil-cement ratio of 0.15. Compressive strength measurements for PFS cement stabilized soil and Portland cement stabilized soil were taken at 7, 28, 60, 75, 90, and 120 days (Fig 3). Generally, strength increased significantly before 28 days. Between 28 and 90 days, strength continued to grow. After 90 days of curing, growth slowed down and became stable. Strength growth was considered mostly complete by 90 days. Evaluating the strength of stabilized waste soil by using the 90-day compressive strength as the design standard was more scientific. However, due to the long 90-day testing period, early strength measurements were often taken to predict the 90-day compressive strength based on the strength development pattern. This study developed a strength prediction model for stabilized soil materials and determined the relevant parameter values, as shown in Eq. (4) [33]. The unconfined compressive strength (*f*) of standard-cured cubic specimens was related to the age-dependent empirical coefficient (*a*). The values for a are 0.4 at 7 days, 1 at 28 days, 1.5 at 90 days, and 1.95 for the final strength. Other parameters include the sand-to-clay ratio (x) and the water-to-cement ratio (y).

$$f=a*\frac{x^{1.35}}{y^{2.95}}\left(correlationcoefficientR=0.987\right)$$ (4)

The compressive strength test results indicated that Portland cement stabilized soil had higher initial strength compared to PFS cement stabilized soil. In the mid-term curing period, the compressive strength of PFS cement stabilized soil exceeded that of Portland cement stabilized soil. In the late curing period, Portland cement stabilized soil experienced very little increase in compressive strength, remaining nearly unchanged. In contrast, PFS cement stabilized soil continued to gain strength. Analysis of the data showed that the average strength of PFS cement stabilized soil was approximately 33.18% higher than that of Portland cement stabilized soil. PFS cement stabilized soil demonstrated better long-term compressive strength performance.

#### 4.2.2 Stress-strain relationship.

**Fig 4:**
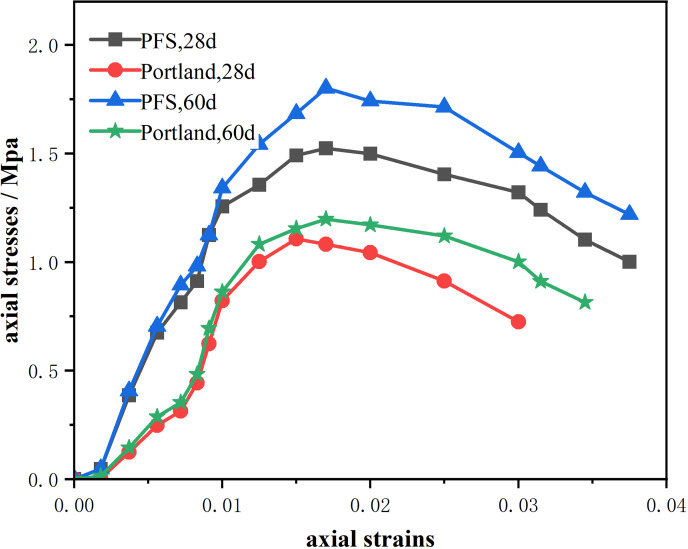
Stress-strain curve.

The stress-strain experiment aimed to compare the mechanical properties of PFS cement and Portland cement in stabilizing waste soil. Conducted was the analysis of the elastic phase, where both PFS cement-stabilized soil and Portland cement-stabilized soil showed a linear stress-strain relationship. Higher elastic modulus was found in PFS cement-stabilized soil, reflecting greater stiffness and stronger resistance to deformation. Shown in Table 5 and Fig 4, PFS cement-stabilized soil had greater load-bearing capacity during the yield phase. For ultimate strength, PFS cement-stabilized soil withstood a larger load before failure. Ductility and toughness were evaluated by strain and the area under the stress-strain curve. Under high-strain conditions, PFS cement-stabilized soil showed greater ductility and toughness. At 60 days, strain reached a higher value while maintaining a high stress level, indicating excellent plastic deformation capability. Failure mode analysis revealed significant strengthening effects in PFS cement-stabilized soil during the hardening phase. At 28 and 60 days, peak stresses were much higher than those in Portland cement-stabilized soil. Stress gradually decreased during the softening phase but remained high, showing better resistance to failure and greater ductility. Portland cement-stabilized soil, in contrast, softened and failed rapidly after reaching maximum stress, demonstrating poor resistance to failure.

**Table 5:** Stress-strain results comparison.

Type	Yield Strength	
	28 d	60 d
PFS cement	1.491 MPa	1.684 MPa
Portland cement	1.0 MPa	1.154 MPa
Type	Ultimate Strength	
	28 d	60 d
PFS cement	1.499 MPa	1.742 MPa
Portland cement	1.082 MPa	1.171 MPa

Comprehensive analysis of the stress-strain performance of PFS cement-stabilized soil and Portland cement-stabilized soil at different curing times revealed significant differences. The yield strength of PFS cement-stabilized soil was 49.1% higher than that of Portland cement-stabilized soil at 28 days and 45.9% higher at 60 days. In terms of ultimate strength, PFS cement-stabilized soil exceeded Portland cement-stabilized soil by 38.5% at 28 days and 48.8% at 60 days. Under a strain condition of 0.03, PFS cement-stabilized soil also showed better ductility. The stress values were 82.5% higher at 28 days and 50.3% higher at 60 days compared to Portland cement-stabilized soil. These data indicated that PFS cement-stabilized soil outperformed Portland cement-stabilized soil in all aspects, particularly in load-bearing capacity, hardening effect, and ductility, showcasing more pronounced advantages.

#### 4.2.3 The relationship of elastic modulus with compressive strength.

The elastic modulus of stabilized soil was determined by the slope of the stress-strain curve under loading [34,35]. The static elastic modulus was obtained from the slope of the line that connected the origin to the point at which σ/ $\sigma_{B}$ equaled 0.4 (40% of the peak stress) [36]. For backfill materials, the elastic modulus was determined by the slope of the line between the origin O and point A on the stress-strain curve. A relationship between the compressive strength and elastic modulus of stabilized soil had been established through various regression models [37,38]. A strong correlation was found using a power-law model, with a correlation coefficient (R^2^) of 0.8637. Based on this, an equation had been developed for the relationship between the 28-day unconfined compressive strength and elastic modulus of the backfill material (Eq. 3). This equation involved the initial tangent modulus $\left(E_{0}\right)$ and the 28-day unconfined compressive strength $\left(f_{c}\right)$.


$$E_{0}=283.86f_{c}0.56\left(R^{2}=0.8637\right)$$ (3)

### 4.3 Durability performance test results

#### 4.3.1 Resistance to dry-wet cycles.

**Fig 5:**
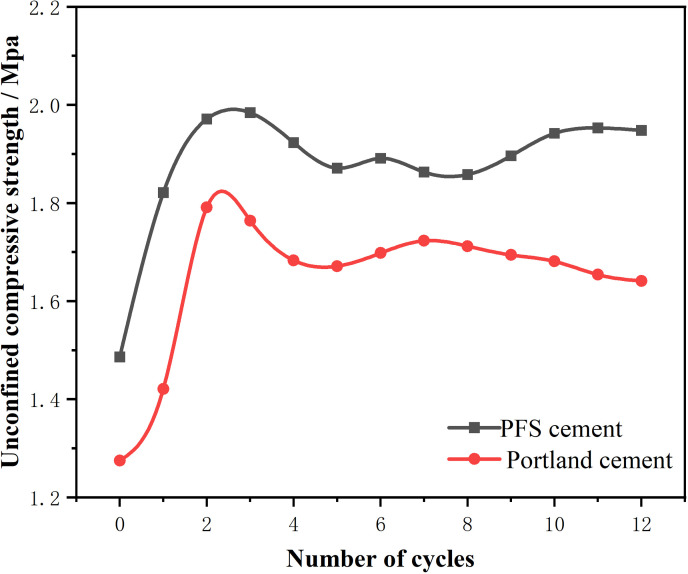
Compressive strength variation under dry-wet cycles.


Comparing the strength variations of PFS cement-stabilized soil and Portland cement-stabilized soil during dry-wet cycles revealed distinct trends. Both stabilized soils exhibited strength improvements after the cycles. Experimental results showed a significant strength increase in PFS cement-stabilized soil after the first dry-wet cycle. This increase resulted from considerable potential for strength enhancement after 28 days of curing. Alternating immersion in water and exposure to high temperatures in the test environment promoted rapid strength growth. After the second cycle, strength initially decreased and then stabilized. In contrast, Portland cement-stabilized soil experienced a gradual decline in strength after undergoing the cycles. Neither stabilized soil, however, experienced structural damage due to the intense dry-wet cycles. Data showed that the strength variation rate of Portland cement-stabilized soil was 5% after 12 cycles, whereas PFS cement-stabilized soil exhibited an 8% variation (Fig 5). Both stabilized soils exhibited a slight increase in strength compared to the 28-day mark, with PFS cement-stabilized soil demonstrating slightly better resistance to dry-wet cycles and durability. The strength range for PFS cement-stabilized soil was between 1.486 MPa and 2.091 MPa. For Portland cement-stabilized soil, the range was between 1.275 MPa and 1.891 MPa. Although both stabilized soils showed fluctuations in strength, the overall fluctuation for PFS cement-stabilized soil was larger. Its later strength values were generally higher than earlier ones, indicating potential for further improvement under specific conditions. In contrast, Portland cement-stabilized soil remained relatively stable but consistently lower than PFS cement-stabilized soil. Overall, the compressive strength of PFS cement-stabilized soil in the dry-wet cycle test was 15.17% higher than that of Portland cement-stabilized soil, demonstrating clear advantages in enhancing material strength and durability. To investigate the strength degradation behavior of the material under wet-dry cycles, regression analysis was used to establish a predictive formula for the strength degradation of recycled backfill material [39]. This formula is based on key parameters such as the sand-to-clay ratio (C/Sa), the water-to-cement ratio (W/So), and the number of cycles (n). The regression coefficients were found to be: b =  -19.91, k1 =  -4.40, k2 =  -10.99, and k3 =  -0.19.

$$f\left(n\right)=b+k_{1}\ln\left(\frac{C}{Sa}\right)+k_{2}\ln\left(\frac{W}{So}\right)+k_{3}\ln\left(n\right),R^{2}=0.83$$ (5)

Under conditions of wet-dry cycling, strength changes in stabilized soil resulted mainly from significant improvements in internal structure during the initial cycles. These improvements depended on the ongoing hydration reaction of cement. Hydration products, such as calcium silicate hydrate, filled internal pores continuously, increasing material density and tensile strength. During early wet-dry cycles, alternating water infiltration and evaporation promoted strength enhancement. In wet conditions, materials maintained a favorable hydration environment, which enhanced structural stability. However, as wet-dry cycling progressed, wet-dry stress accumulated. Especially during drying, internal stress exceeded the tensile strength of the material, leading to crack formation and propagation. Repeated wet-dry stress deteriorated the pore structure, gradually increased porosity, and ultimately reduced material strength. Compared to ordinary Portland cement, PFS cement showed superior performance under wet-dry cycling. Fly ash and slag in PFS cement participated in secondary hydration reactions. These reactions produced denser calcium silicate hydrate and calcium aluminate hydrate, further reducing internal porosity and enhancing crack resistance and durability. Therefore, under long-term wet-dry cycling, PFS cement demonstrated excellent resistance to degradation, significantly outperforming Portland cement.

#### 4.3.2 Resistance to freeze-thaw cycles.

During the initial freeze-thaw cycles, the strength of both stabilized waste soils gradually increased. The primary reason was the expansion stress generated by water within the stabilized soil when freezing. As the strength of the stabilized soil increased, the tensile strength of the material also improved. The enhanced tensile strength allowed the material to resist stress exerted by expansion pressure on pore walls, preventing irreversible structural damage such as microcracks and improving frost resistance. However, with continued freeze-thaw cycling, the cohesion between soil particles gradually became less than the frost heave force, leading to a significant reduction in strength. Strength changes during freeze-thaw cycles were mainly influenced by frost heave stress. When the material was in a saturated state and subjected to low temperatures, internal water froze, expanded in volume, and exerted expansion pressure on the pore walls. As the number of cycles increased, the destructive effects of expansion pressure on pore structures accumulated. When expansion pressure exceeded the tensile strength of the material, microcracks began to form within the material. Cracks further expanded and connected during subsequent freeze-thaw cycles, gradually weakening the material’s strength. Additionally, during thawing, water entered the cracks, increasing the water content in the pores. Upon the next freezing cycle, the water volume expanded again, further exacerbating the structural damage. With the repeated occurrence of this process, material strength gradually diminished, ultimately leading to irreversible structural failure.

**Fig 6:**
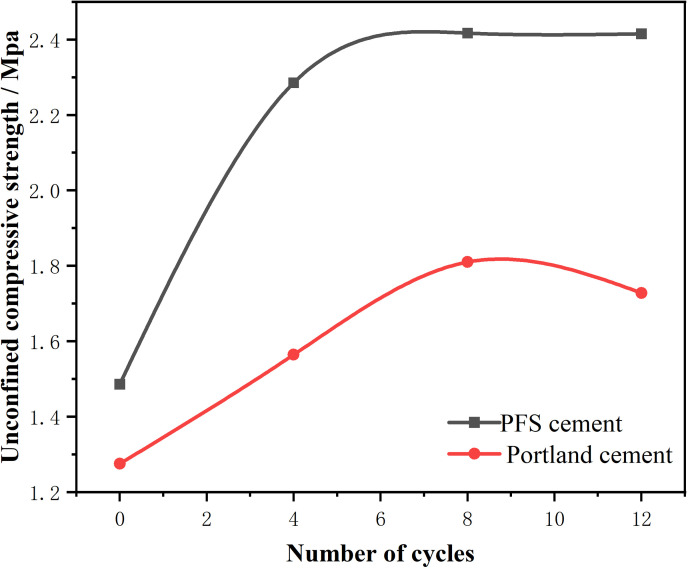
Compressive strength variation under freeze-thaw cycles.


The analysis indicated that as the number of freeze-thaw cycles increased, the strength of PFS cement-stabilized soil gradually improved. After the sixth cycle, the strength stabilized around 2.4 MPa (Fig 6). In comparison, Portland cement-stabilized soil also showed an increase in strength during the initial cycles. However, it peaked at 1.7-1.8 MPa after the eighth freeze-thaw cycle and then began to decline gradually. Both types of stabilized soils met the required performance in freeze-thaw cycle tests. Portland cement-stabilized soil, however, deteriorated with repeated cycles. PFS cement-stabilized soil maintained a strength approximately 29.95% higher than Portland cement-stabilized soil, demonstrating superior durability and stability. Hydration products produced by PFS cement not only made the material denser but also improved crack resistance. Improved crack resistance enhanced the material’s ability to resist expansion stress caused by freezing water during freeze-thaw cycles. As water froze and expanded within the material, the strength and dense structure of PFS cement effectively dispersed and resisted expansion stresses, reducing the formation of internal microcracks. In contrast, Portland cement had a looser pore structure and fewer hydration products, which led to easier microcrack propagation under frost heave pressure during freeze-thaw cycles, accelerating the decline in strength. The density and crack resistance of PFS cement contributed to its superior performance in freeze-thaw cycles, delaying material degradation and enhancing resistance to freeze-thaw damage. To explore the strength degradation under freeze-thaw cycles, regression analysis was also used to establish a predictive formula for the freeze-thaw cycle strength loss [40]. The formula uses the same key parameters: sand-to-clay ratio (C/Sa), water-to-cement ratio (W/So), and number of cycles (n). The regression coefficients for this model are: b =  6.68, k1 =  27.67, k2 =  -23.64, and k3 =  0.02.

$$f\left(n\right)=b+k_{1}\ln\left(\frac{C}{Sa}\right)+k_{2}\ln\left(\frac{W}{So}\right)+k_{3}\ln\left(n\right),R^{2}=0.93$$ (6)

In freeze-thaw cycle experiments, the compressive strength alterations of materials under varying numbers of cycles were noted. For frozen saturated soil, temperature and strain rate notably affected its compressive strength. Experimental outcomes demonstrated that as freeze-thaw cycles rose, the strength of PFS cement-stabilized soil grew incrementally, with more prominent freeze-thaw performance at low temps. During freezing, the expansion stress from water expansion at low temps was resisted by PFS cement’s dense structure and high strength, enhancing its frost resistance. Regarding the UCS of frozen saturated clay, temperature changes and strain rate effects were conspicuous. Compared to Portland cement-stabilized soil, PFS cement-stabilized soil had better frost resistance, higher compressive strength, and less strength fluctuation after freeze-thaw cycles[41]. In contrast, Portland cement-stabilized soil had significant strength changes during freezing and declined after freeze-thaw cycles. This signified that temperature and freeze-thaw strain rate had substantial impacts on soil UCS, and PFS cement’s frost and crack resistance made it superior [42]. Further analysis showed that freezing temperature changes had a major influence on material compressive strength. As freeze-thaw cycles advanced, lower freezing temps led to greater UCS value fluctuations, and as temp rose, material strength weakened [43]. At low strain rates, the negative effect of freeze-thaw cycles on strength was reduced, while high strain rates worsened strength decline. In general, PFS cement’s excellent performance was closely tied to its dense pore structure and low water permeability, which mitigated the adverse effects of temperature and strain rate on freeze-thaw resistance.

### 4.4 Comparative analysis of components

Table 6 compared the main chemical components of PFS cement and Portland cement. Results showed significant differences in the composition of the two types. Lower was the CaO content in PFS cement, while SiO₂, Al₂O₃, and SO₃ contents were significantly higher than those in Portland cement. High SiO₂ and Al₂O₃ contents contributed to the formation of more C-S-H gel and ettringite, enhancing the durability and heavy metal stabilization of PFS cement. Although CaO content was lower in PFS cement, possibly affecting strength, high Al₂O₃ and SiO₂ contents provided significant advantages in chemical stability and erosion resistance. These differences indicated that PFS cement had unique advantages in heavy metal stabilization and chemical resistance applications.

**Table 6:** Major chemical components of PFS cement and Portland cement.

Components	CaO	SiO_2_	Al_2_O_3_	SO_3_	MgO	Fe_2_O_3_	Na_2_O	TiO_2_	others
PFS Cement	35.19	28.23	14.67	8.55	5.84	3.05	0.93	0.78	2.70
Portland cement	59.60	18.28	5.76	5.26	6.12	2.70	0.19	0.35	1.70

## 5. Discussion

Stabilization of construction waste soil with Portland cement involved multiple reactions. Ion exchange, flocculation, hardening, carbonation, and crystallization occurred during the process. Water acted both as a reactant and a medium for these reactions. Not all water participated fully. Some remained in the stabilized soil, creating residual pores over time and weakening the overall strength. Therefore, the development of new PFS cement aimed to enhance mechanical and durability performance of stabilized soil. Steel slag, fly ash, and phosphogypsum in PFS cement contributed through hydration, ion exchange, pozzolanic reaction (cementation), flocculation, precipitation, polymerization, oxidation, and carbonation. Expansion crystals and cementitious particles were produced. These reactions modified the load-bearing framework and filled voids. The reactions also altered physical and mechanical properties, transforming loose and weak soil into a cohesive, high-strength stabilized material.

### 5.1 Physicochemical reactions of steel slag in PFS cement enhance the strength of stabilized soil

**Fig 7:**
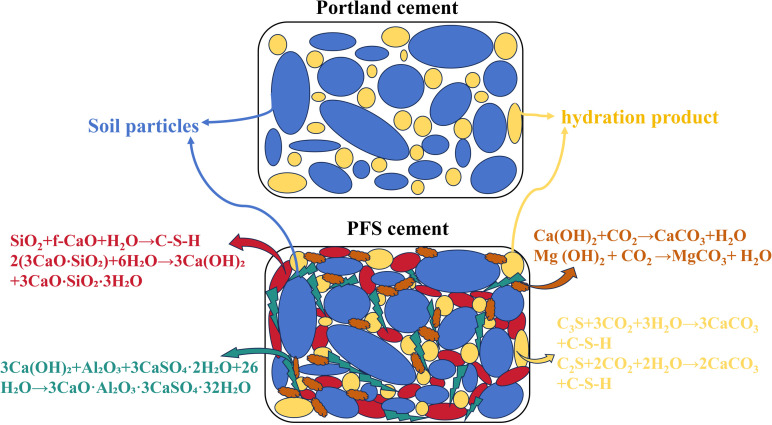
Hydration mechanism of steel slag.

Steel slag, as a weak hydraulic material, exhibited two main phases in cement-stabilized soil. In the early stage, steel slag primarily existed as granules, providing friction and cohesion between particles [44]. In the later stage, various reactions enhanced the performance of stabilized soil. C₃S and C₂S in steel slag generated a large amount of Ca(OH)₂ during hydration. Additionally, f-CaO in steel slag reacted with oxygen and free water, forming Ca(OH)₂ [45]. Newly formed Ca(OH)₂ reacted with SiO₂, Fe₂O₃, and Al₂O₃ in steel slag, producing C-H-S, C-H-F, and C-H-A compounds [46]. Hydration of steel slag also involved carbonation reactions with CO₂, where CaO and MgO formed CaCO₃ and MgCO₃ [47]. These new compounds embedded into C-S-H structures, further enhancing the microstructure and mechanical strength. Besides oxidation, f-CaO also reacted with active SiO₂ in fly ash, producing more stable C-S-H gel [48]. SiO₂ lowered the Ca/Si ratio in the C-S-H structure, which resulted in a denser stabilized soil [49]. Phosphogypsum in PFS cement accelerated the hydration reaction and reacted chemically with steel slag. In the sulfate-rich environment provided by phosphogypsum, the small amount of C₃A in steel slag reacted with Ca(OH)₂ to produce C₄AH₁₃. C₄AH₁₃ then underwent secondary hydration with gypsum, forming calcium aluminate sulfate hydrate (C-A-S-H) [50]. These products, in the presence of a large amount of gypsum, formed ettringite. The rod-like ettringite crystals interlocked between different particles, enhancing the contact between solid phases and greatly promoting material strength [51]. C-S-H gel and ettringite crystals acted as binders, holding these particles together, making the structure more compact and further improving mechanical strength. This reaction process not only improved the chemical stability of the material but also enhanced its physical properties. The gels and crystals produced by the hydration reaction of steel slag formed a cohesive system through cementation. This system filled and connected the gaps between aggregates, compacting both the interparticle and intraparticle voids. Such a system provided anti-swelling cementation strength to the stabilized soil, significantly improving the strength, stability, and crack resistance of the waste-stabilized material. The results demonstrated the advantage of steel slag in stabilizing waste soil.(Fig 7)

### 5.2 Fly ash in PFS cement enhanced durability of stabilized soil

Fly ash consisted of fine particles, generally spherical and hollow, with an amorphous glassy nature. SiO₂, Al₂O₃, Fe₂O₃, and unburned carbon composed the primary elements [52]. In soil stabilization, fly ash played an essential role due to unique physical and chemical properties [53]. Main mechanisms included physical filling, ion exchange, and pozzolanic reaction. Significant improvements were observed in mechanical properties and chemical stability of stabilized soil. Fine particles and glassy silicate in fly ash contributed to filling and reactivity, which enhanced soil density and load-bearing capacity [54]. Workability also improved, with increased slurry fluidity. Slurry filled the voids between soil particles, coating and lubricating soil grains [55]. As a result, cohesion and plasticity of the stabilized soil mixture were enhanced.

**Fig 8:**
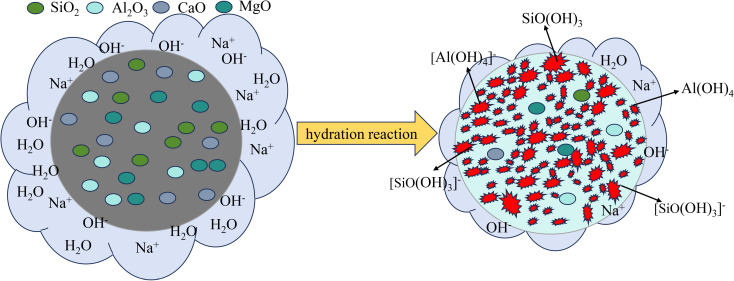
Hydration mechanism of fly ash.

The use of fly ash in stabilized soil not only enhanced physical and mechanical properties but also improved chemical stability. Aluminosilicate components in fly ash underwent ion exchange reactions with soil cations [56]. These reactions immobilized heavy metal ions, reducing their mobility and toxicity, which improved soil environmental stability [49]. The alkaline nature of fly ash neutralized acidic substances in the soil [57]. This neutralization helped regulate soil pH and improve the chemical environment. Hydroxyl groups on the surface of fly ash [58] provided good permeability to stabilized soil when in a loose state. The pozzolanic effect of fly ash [57] contributed to the late-stage strength growth of stabilized waste material. An NaOH alkali activator caused reactive glassy silica and alumina in fly ash to react with cement paste. The reaction formed calcium hydroxide and magnesium hydroxide. These hydroxides then reacted with silicates and aluminates in soil, producing calcium silicate hydrate (C-S-H) and calcium aluminate hydrate (C-A-H) [59]. The resulting products formed gel-like cementitious substances between soil particles, filling the hydrolysis layer. This formation enhanced cohesion, impermeability, and abrasion resistance of the soil, leading to improved structural integrity. Stabilized soil initially exhibited low early strength. Strength significantly increased at later stages due to ongoing pozzolanic activity. Adding fly ash reduced the heat of hydration, minimized cracking, and improved soil density and impermeability. Secondary hydration reactions [60] played a crucial role in enhancing the strength of stabilized waste material, especially under harsh conditions. The enhanced structure improved the material’s resistance to damage. The composite effect of fly ash enhanced the basic physical and mechanical properties of the soil. The addition of fly ash also improved environmental adaptability and long-term stability through various chemical reactions. The improvements demonstrated the irreplaceable role of fly ash in enhancing the durability of stabilized waste material. In the later stages of the reaction, PFS cement showed significant strength gains. Consequently, stabilized soil maintained consistently high strength levels (Fig 8).

## 6. Conclusions

A novel PFS cement was proposed in this paper, and its application in trench backfilling projects in Yantai, Shandong Province, China, was investigated through field testing. The formulation of PFS cement from solid waste materials was innovative, and significant breakthroughs were achieved in the enhancement of soil strength. The main conclusions are as follows:

(1) Compressive strength test results indicated that PFS cement had a compressive strength about 33.18% higher than Portland cement at 90 days. Yield strength at 28 and 60 days for PFS cement was 49.1% and 45.9% higher, respectively. Maximum stress was 38.5% and 48.8% higher than Portland cement at the same intervals.
(2) Wet-dry cycle tests showed that, after 12 cycles, compressive strength of PFS cement-stabilized soil was 15.17% higher compared to Portland cement. Freeze-thaw cycle tests indicated that PFS cement-stabilized soil maintained a high compressive strength level, 29.95% higher than Portland cement after multiple cycles.
(3) C₃S and C₂S in steel slag formed large quantities of Ca(OH)₂ during hydration, which reacted with SiO₂, Fe₂O₃, and Al₂O₃ to form C-H-S, C-H-F, and C-H-A structures, enhancing the microstructure of stabilized soil. CaO and MgO reacted with CO₂ to produce CaCO₃ and MgCO₃, further embedding into C-S-H structures, thus increasing strength. Fly ash reacted with Ca(OH)₂ through pozzolanic effects, producing calcium silicate hydrate and calcium aluminate hydrate, forming a cementitious material that filled interparticle pores, increased soil cohesion and density, and reduced the Ca/Si ratio, resulting in a more compact stabilized soil structure.

The results demonstrate that PFS cement can completely replace Portland cement, meeting and exceeding the strength requirements for stabilizing waste soil, while offering superior durability and environmental adaptability. When used as a stabilizing agent, the treated soil can be applied in various construction scenarios, including underground engineering backfilling, trench backfilling, roadbed backfilling, and bridge abutment backfilling. Moreover, PFS cement, made primarily from solid waste materials, significantly reduces carbon emissions, providing a more environmentally friendly solution for soil stabilization. Future research can further explore the application potential of PFS cement in stabilizing special waste soils and complex soil conditions, as well as its expanded use in other construction fields.

## Supporting information

S1 Fig Result of unconfined compressive strength. (PDF)

S2 Fig Result of stress-strain curve. (PDF)

S3 Fig Result of compressive strength variation under dry-wet cycles. (PDF)

S4 Fig Result of compressive strength variation under freeze-thaw cycles. (PDF)
