# Supplementary material for: Comparison of the stabilized waste soil properties and stabilization mechanism of phosphogypsum-fly ash-steel slag based cement versus Portland cement
Source: PLoS One. 2025 Jun 3;20(6):e0318862. doi: 10.1371/journal.pone.0318862 (PMC12132936; doi:10.1371/journal.pone.0318862)
Supplement: S1 Fig — (PDF) [file pone.0318862.s001.pdf]

Supporting Information

Figure 3 Unconfined Compressive Strength

The raw data for unconfined compressive strength, corresponding to Figure 3 in the manuscript.

Time (days)	PFS Cement (Mpa)	Portland Cement (Mpa)
7	0.718	0.812
28	1.65	1.284
60	2.12	1.506
90	2.63	1.712
120	3.12	2.048
