# Supplementary material for: Comparison of the stabilized waste soil properties and stabilization mechanism of phosphogypsum-fly ash-steel slag based cement versus Portland cement
Source: PLoS One. 2025 Jun 3;20(6):e0318862. doi: 10.1371/journal.pone.0318862 (PMC12132936; doi:10.1371/journal.pone.0318862)
Supplement: S2 Fig — (PDF) [file pone.0318862.s002.pdf]

Supporting Information

Figure 4 Stress-Strain Curve

The raw data for the stress-strain curves, corresponding to Figure 4 in the manuscript,

Axial strains	Axial stressed/ Mpa			
	PFS 28d	Portland 28d	PFS 60d	Portland 60d
0	0	0	0	0
0.0018	0.047	0.004	0.047	0.014
0.0037	0.386	0.124	0.406	0.144
0.0056	0.674	0.247	0.704	0.287
0.0072	0.814	0.313	0.894	0.353
0.0083	0.912	0.443	0.982	0.483
0.0091	1.124	0.624	1.124	0.694
0.01	1.256	0.821	1.341	0.862
0.0125	1.356	1.107	1.542	1.082
0.015	1.491	1.002	1.684	1.154
0.017	1.524	1.043	1.801	1.198
0.02	1.499	1.082	1.742	1.171
0.025	1.404	0.912	1.714	1.12
0.03	1.321	0.724	1.504	1.001
0.0315	1.241		1.442	0.911
0.0345	1.103		1.321	0.814
0.0375	1.001		1.219	
