# Supplementary material for: Comparison of the stabilized waste soil properties and stabilization mechanism of phosphogypsum-fly ash-steel slag based cement versus Portland cement
Source: PLoS One. 2025 Jun 3;20(6):e0318862. doi: 10.1371/journal.pone.0318862 (PMC12132936; doi:10.1371/journal.pone.0318862)
Supplement: S3 Fig — (PDF) [file pone.0318862.s003.pdf]

Supporting Information

Figure 5 Compressive Strength Variation under Dry-Wet Cycles

The raw data for the compressive strength results under wet-dry cycling, corresponding to Figure 5 in the manuscript.

Number of cycles	PFS cement / Mpa	Portland cement / Mpa
0	1.486	1.275
1	1.821	1.421
2	1.971	1.891
3	2.901	1.764
4	1.923	1.683
5	1.871	1.671
6	1.891	1.698
7	1.863	1.723
8	1.858	1.712
9	1.896	1.694
10	1.942	1.681
11	1.953	1.654
12	1.948	1.641
