# Supplementary material for: Comparison of the stabilized waste soil properties and stabilization mechanism of phosphogypsum-fly ash-steel slag based cement versus Portland cement
Source: PLoS One. 2025 Jun 3;20(6):e0318862. doi: 10.1371/journal.pone.0318862 (PMC12132936; doi:10.1371/journal.pone.0318862)
Supplement: S4 Fig — (PDF) [file pone.0318862.s004.pdf]

Supporting Information

Figure 6 Compressive Strength Variation under Freeze-Thaw Cycles

The raw data for the compressive strength results under freeze-thaw cycling, corresponding to Figure 6 in the manuscript.

Number of cycles	PFS cement / Mpa	Portland cement / Mpa
0	1.486	1.275
4	2.21	1.543
8	2.394	1.824
12	2.385	1.731
